# *Claudin h* Is Essential for Hair Cell Morphogenesis and Auditory Function in Zebrafish

**DOI:** 10.3389/fcell.2021.663995

**Published:** 2021-05-11

**Authors:** Jie Gong, Peipei Qian, Yuebo Hu, Chao Guo, Guanyun Wei, Cheng Wang, Chengyun Cai, Haibo Wang, Dong Liu

**Affiliations:** ^1^Nantong Laboratory of Development and Diseases, School of Life Sciences, Co-innovation Center of Neuroregeneration, Key Laboratory of Neuroregeneration of Jiangsu and MOE, Nantong University, Nantong, China; ^2^Shandong Provincial ENT Hospital, Cheeloo College of Medicine, Shandong University, Jinan, China

**Keywords:** *claudin h*, otic vesicle, hearing loss, vestibular dysfunction, hair cell, zebrafish

## Abstract

Hereditary hearing loss caused by defective hair cells is one of the most common congenital diseases, whose nosogenesis is still unclear because many of the causative genes remain unidentified. Claudins are one kind of transmembrane proteins that constitute the most important components of the tight junctions and paracellular barrier and play important roles in neurodevelopment. In this study, we investigated the function of *claudin h* in morphogenesis and auditory function of the hair cell in zebrafish. The results of *in situ* hybridization showed that *claudin h* was specifically localized in the otic vesicle and neuromasts in zebrafish embryos. The deficiency of *claudin h* caused significant reduction of otic vesicle size and loss of utricle otolith. Moreover, the startle response and vestibulo-ocular reflex experiments revealed that loss of *claudin h* led to serious hearing loss and vestibular dysfunction. Importantly, the confocal microscopy observation found that compared to the control zebrafish, the *claudin h* morphants and mutants displayed significantly reduced the number of cristae hair cells and shortened kinocilia. Besides, the deficiency of *claudin h* also caused the loss of hair cells in neuromasts which could be rescued by injecting *claudin h* mRNA into the mutant embryos at one cell stage. Furthermore, the immunohistochemistry experiments demonstrated remarkable apoptosis of hair cells in the neuromasts, which might contribute to the loss of hair cells number. Overall, these data indicated that *claudin h* is indispensable for the development of hair cells, vestibular function, and hearing ability of zebrafish.

## Introduction

Hair cells are one of the sensory cells in the auditory epithelium of the mammalian inner ear which are necessary for transforming sound vibration and mechanical forces including gravity into neural signals that can be interpreted by the brain (Goutman et al., 2015). In mammals, there are two different hair cells classified as outer hair cells and inner hair cells. Outer hair cells that have three rows of cells can mechanically amplify sound-induced vibrations to increase the responsiveness of sensory epithelium, while inner hair cells have only one row of cells and are functional in transmitting the signals to the spiral ganglion neurons (Ma et al., 2018). Therefore, the number and function of hair cells are crucial for hearing (Liu et al., 2019; Qi et al., 2019; He et al., 2020). Hearing loss could be caused by different reasons including genetic factors, aging, infectious diseases, ototoxic drugs, and noise exposure (Zhu et al., 2018; Gao et al., 2019), among which the genetic factors contribute the most by leading to hereditary and progressive hearing loss. To date, the mechanisms of hair cell damage mainly include mechanical shearing forces and oxidative damage to hair cells, which eventually induce apoptotic cell death in hair cells (Liu et al., 2016; Ding et al., 2020; Zhou et al., 2020). The lower vertebrates have the hair cell regeneration ability though life times; while in mammals, although neonatal mice cochlea have limited hair cell regeneration ability, this regeneration ability is rapidly reduced with age (Wang et al., 2015; Zhang et al., 2020).

A typical characteristic of biological development is to separate the compositionally distinct extracellular fluids by sealing off the paracellular spaces (Madara, 1998). The essential structure of this seal formed with epithelial sheets is the continuous band-like networks of neighboring cells that are known as tight junctions (Nakano et al., 2009). Tight junctions that circumscribe the apical end of the cell can make the membranes of the adjacent cells closely connected and thereby split the cells into the basolateral and apical region, and then form a barrier to restrict paracellular permeability of ions and solutes (Dejana, 2004). In recent years, several integral membrane proteins have been identified to be involved in the function of the tight junctions and the specific expression of these proteins in different tissues may account for various “tightness” of tight junctions (Van Itallie and Anderson, 2004; Li et al., 2018). Nowadays, at least three types of transmembrane proteins, occludin, claudin family, and the junction adhesion molecule family, are reported to compose the tight junctions by interacting with the cytoskeleton and other membrane-associated proteins (Furuse et al., 1993; Martin-Padura et al., 1998; Tsukita and Furuse, 2000).

Claudins have been reported to be the primary components of the tight junctions in many tissues to form the backbone of tight junction strands (Tipsmark et al., 2008). The claudins are conserved in different species for the membrane topology with two extracellular loops, one N-terminal cytoplasmic domain and one C-terminal cytoplasmic domain (Li et al., 2011). The extracellular loops are essential for forming the paracellular barrier structure by polymerizing the intercellular claudins into strands, while the C-terminal cytoplasmic domains of claudins can coordinate with the scaffolding proteins to link the tight junctions to the actin cytoskeleton (Wen et al., 2004; Krause et al., 2008). The epithelia of mammalian nephric tubules utilize abundant tight junctions to transport ions selectively between the intercellular regions (Gong and Hou, 2017). Many different claudins are reported expressing in different segments of nephric tubules and are necessary for kidney function as selective paracellular pores or barriers to regulate the diffusion of different ions and water via tight junctions (Van Itallie et al., 2006; Angelow et al., 2007; Milatz et al., 2010; Li et al., 2011). For example, it has been reported that in the porcine kidney epithelial cell line, Claudin-7 can form a paracellular barrier to Cl-, but serve as a paracellular channel for Na^+^ (Alexandre et al., 2005). In addition, there are some claudins which are not merely expressed in tight junctions, but also in some other cellular parts play important roles in embryonic development (Gregory et al., 2001; Kollmar et al., 2001).

The zebrafish has become an excellent model organism in biological research to investigate otic vesicle and neural development which are closely related to the human diseases. The greatest advantage of using zebrafish for research is the convenience for recording and tracking of the developmental process by imaging (Downes and Granato, 2004; Bandmann and Burton, 2010). Moreover, the otic vesicle of zebrafish separates the distinct paracellular fluids compositionally and therefore becomes a great model for investigating the function of claudins and tight junctions on hearing and balance. Recently, loss of both *claudin7b* and *claudin j* was found to lead to the abnormal otolith formation and hair cell function and further cause the inner ear dysfunction in the zebrafish (Hardison et al., 2005; Li et al., 2018). However, the functions of other claudins in the inner ear remain unclear, and thus it promoted us to establish a loss of function model to study the role of claudins in hearing function.

In this study, we found that *claudin h* (*claudin 3* in mammals) were expressed in the otic vesicle and neuromasts of zebrafish embryos by *in situ* hybridization. Loss of function treatments by either morpholino injection or CRISPR-cas9 could both cause hearing loss and vestibular dysfunction. We further found that these dysfunctions might be caused by abnormal otolith formation, hair cell loss, and otic vesicle morphological defects. Moreover, *claudin h* deficiency could induce hair cell apoptosis, which explained the decrease in number of the hair cells. In summary, our study proved that *claudin h* was essential for the formation of hair cell and otoliths and the normal hearing function of the inner ear in zebrafish.

## Materials and Methods

### Zebrafish Husbandry

The zebrafish embryos and adults were maintained in the zebrafish Center of Nantong University under conditions following our previous protocols (Gong et al., 2020). Wild-type (AB) control and *Tg(Brn3c:GFP)* transgenic zebrafish whose hair cells were labeled by GFP were used in this study (Xiao et al., 2005).

### Whole Mount *in situ* Hybridization

Whole-mount *in situ* hybridization (WISH) was performed according to our previous procedures (Huang et al., 2013). A 409 bp cDNA fragment of *claudin h* was amplified from zebrafish embryo cDNA library with specific primers (Table 1) and inserted into pGEM-T-easy vector. Digoxigenin-labeled antisense probes were synthesized with the linearized pGEM-T inserting with *claudin h* construct by DIG-RNA labeling kit (Roche, Switzerland). Zebrafish embryos without pigment at different developmental stages were collected and fixed with 4% PFA overnight at 4°C. After incubated with the probe overnight, an alkaline phosphatase-conjugated antibody against digoxigenin and AP-substrate NBT/BCIP solution (Roche, Switzerland) was used to detect the digoxigenin-labeled RNA probe.

**TABLE 1:** Summary of primers used.

**Primers**	**Primer sequence (5′-3′)**	**Purpose**
*Cldnh*-F	CGATGGGACTGGAAATTGGG	Riboprobe amplification for *Cldnh*
*Cldnh*-R	CCCAGGCTGAAGGAATGAGA	Riboprobe amplification for *Cldnh*
*Cldnh*-gRNA	GGTGTGATGATCTCCGTCAT	Fragment amplification for gRNA
*Cldnh*-gRNA F	ATGTGGCGTGTCTCGGCCTT	Fragment amplification for *Cldnh* gRNA
*Cldnh*-gRNA R	ACAGCATGGCTCCTCCAATG	Fragment amplification for *Cldnh* gRNA

### Morpholino and mRNAs Injections

Splicing-blocking Morpholino (5′- ATGAATGTCATTTACCAA GTGTCGA -3′) that was specific for *claudin h* gene was synthesized by Gene Tools. *Tg(Brn3c:GFP)* zebrafish were naturally mated to obtain embryos for microinjection. The Morpholino was diluted to 0.3 mM with RNase-free water and injected into one cell stage embryos and then raised in E3 medium at 28.5°C for imaging.

### Immunohistochemistry

The larvae at 96 hpf were fixed with 4% PFA for 2 h at room temperature and then washed with PBST for 30 min followed with antigen retrieval at 98°C for 15 min. After washed three times with PBST, the larvae were incubated in blocking solution for 1 h and then were transferred to the primary antibody solutions (anti-GFP, 1:1000 dilution; Abcam, Cambridge, United Kingdom and anti-cleaved caspase-3, 1:500 dilution; Cell Signaling Technology Inc., Danvers, MA, United States) overnight at 4°C. Then, the Alexa Fluor 488 and Alexa Fluor 647 conjugated secondary antibodies were added to larvae at a dilution of 1:500 in blocking solution and incubated for 1 h at room temperature after washed three times with PBST. Nuclei were labeled with 4,6-diamidino-2-phenylindole (DAPI) (1:1000 dilution; Invitrogen, Carlsbad, CA, United States) for 20 min at room temperature and then mounted for imaging.

### sgRNA/Cas9 mRNA Synthesis and Injection

Cas9 mRNA was obtained by *in vitro* transcription with the linearized plasmid pXT7-Cas9 by the mMESSAGE mMACHIN Kit according to the manufacturer’s instruction. For the sgRNA synthesis, a forward primer that contained the *claudin h* specific primers and a universal reverse gRNAR primer (Table 1) were used for sgDNA amplification with pT7 plasmid as the template, and then transcribed into sgRNA using the MAXIscript^®^ kits according to the manufacturer’s instruction. One-cell stage zebrafish embryos were injected with 2–3 nl solution containing 250 ng/μl Cas9 mRNA and 15 ng/μl sgRNA. At 72 and 96 hpf, zebrafish embryos that developed normally were randomly sampled for the confocal imaging and genomic DNA extraction to determine the mutations by DNA sequencing.

### mRNA Rescue Experiments

The *claudin h* mRNA was transcribed *in vitro* using linearized artificial PCS2+ vector with the *claudin h* open reading frame cDNAs by the mMESSAGE mMACHIN Kit according to the manufacturer’s instruction (Ambion, United States). After purified using RNeasy Mini Kit (Qiagen, Germany), 2 nl capped mRNA was co-injected with *claudin h* Mo into one-cell stage embryos.

### Vestibulo-Ocular Reflex (VOR) Testing

The zebrafish larva was gently mounted in the larva-shaped chamber in a dorsal-up position with the tail glued by 5% methylcellulose and covered with a piece of glass coverslip on the chamber. After adding E3 embryo media in the head region, the chamber unit was then mounted on a device for quantifying linear vestibulo-ocular reflex (VOR) from Southern University of Science and Technology (Sun et al., 2018). After aligning the larval eyes to the center of the infrared camera, the platform started to rotate back and forth around a horizontal axis at a speed of 30 rpm, and the VOR was recorded by the camera.

### Acoustic Startle Reflex

About 20 larvae were put in a thin layer of culture media in a petri dish attached to mini vibrator. The response of larvae to sound stimulus (a tone burst 9 dB re. m s^–2^, 600 Hz, for 30 ms) generated by the vibrator was recorded from above by an infrared camera over a 6 s period. The mean moving distance and peak speed were used to quantify the startle response.

### Microscopy and Statistical Analysis

After being anesthetized with tricaine, the zebrafish embryos were mounted in 0.8% low melt agarose, and then photographed by Leica TCS-SP5 LSM confocal microscope. For the *in situ* hybridization, photographs were taken using an Olympus stereomicroscope MVX10. Statistical analyses were performed by one-way analysis of variance (ANOVA), student’s *t*-test or chi-squared test, and *P* values < 0.05 were considered statistically significant.

## Results

### *Claudin h* Gene Is Evolutionarily Conserved and Expressed in the Otic Vesicle and Hair Cells

**FIGURE 1:**
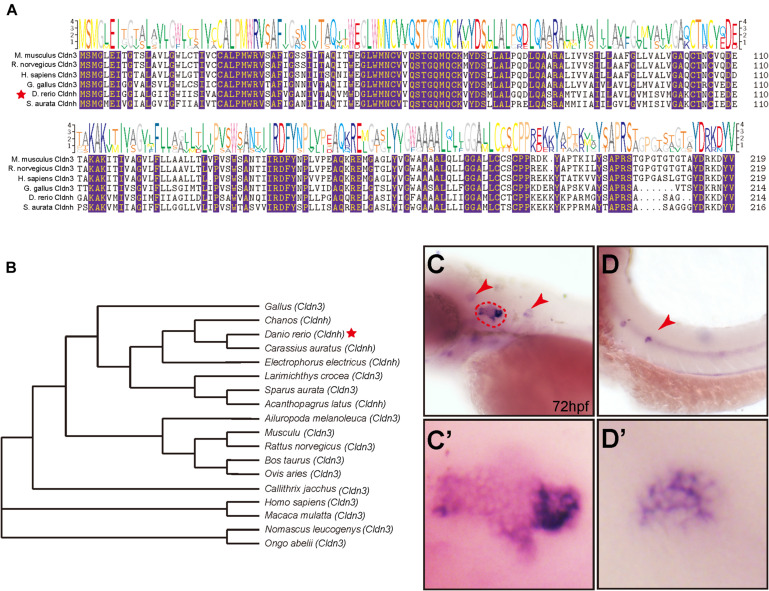
The phylogenetic and expression analysis of zebrafish *claudin h*. **(A)** The alignment of *claudin h* amino acid sequences from different species, and the identical aa residues among all the aligned sequences are labeled with color. **(B)** Phylogenetic analysis of *claudin h*. Neighbor-joining tree was produced with the Mega 5.0 software and the red star marked the zebrafish. **(C,D)** At 72 hpf, the *in situ* hybridization signal of *claudin h* is localized in the otic vesicle and neuromast. The red dotted line marked the boundary of the otic vesicle and the red arrow head marked the neuromast in the head and posterior lateral line. **(C′,D′)** The magnified figure of the positive signals in otic vesicle and neuromast line.

To determine the relationships of zebrafish *claudin h* with other homologous genes, the multiple alignments of *claudin h* from different species were performed and the phylogenetic tree of the *claudin h* was constructed using the Neighbor-joining method. As shown in Figure 1A, the zebrafish *claudin h* gene had significantly high amino acid sequence similarities to other species. The NJ tree of *claudin h* showed that the *claudin h* from osteichthyes and birds were clustered in a separate clade from rodents and primates *claudin h* (Figure 1B).

To investigate the role of *claudin h* during embryonic development, we tested the expression profile of *claudin h* in zebrafish by WISH with a digoxigenin-labeled *claudin h* probe. The results showed that at 24 hpf, *claudin h* was expressed in the otic vesicle, posterior lateral line primordium and pronephros (Supplementary Figure 1A), while from 36 hpf, the gene started to be localized in lateral line neuromasts (Figures 1D,D’ and Supplementary Figure 1B). Besides, the *claudin h* was also detected in the neuromasts of the head with continued development (Figures 1C,C’ and Supplementary Figure 1). Together these results suggested that *claudin h* might be vital for the development of otic vesicle and neuromast.

### Deficiency of *Claudin h* Caused Developmental Defects of Otic Vesicle and Otoliths

**FIGURE 2:**
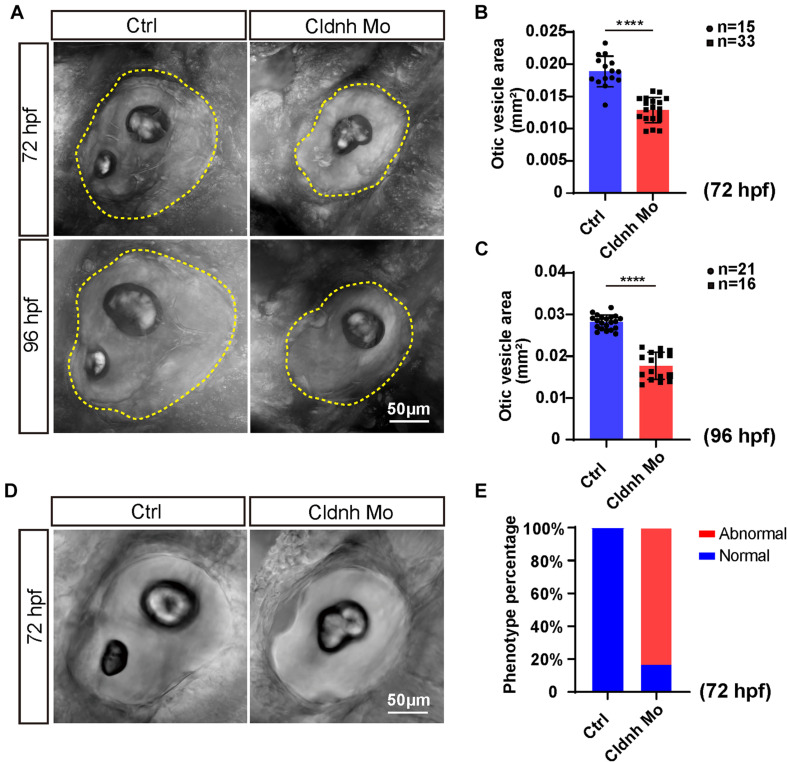
Loss function of *claudin h* caused the defects of otic vesicle and otoliths. **(A,D)** Imaging analysis of otic vesicle and otoliths in control and *claudin h* knocking down groups at 72 and 96 hpf. The yellow dotted line marked the boundary of the otic vesicle. Scale bar = 50 μm. **(B,C)** The statistical analysis of otic vesicle area in the control and *claudin h* morphants at 72 and 96 hpf. **(E)** Quantification of zebrafish embryos with abnormal otolith (defects in both number and shape of otoliths: *claudin h* morphants lost the utricle otolith or had a unnormal saccular otolith). Each bar represents the mean ± SE. Values with **** above the bars are significantly different (*P* < 0.0001).

Since a significant expression of *claudin h* was found in the otic vesicle, we examined the morphology of otic vesicle and otolith in the *claudin h* knocking down zebrafish by confocal microscopy at 72 and 96 hpf to investigate whether *claudin h* regulates the formation of otic vesicle. The results showed that although the morphants had no remarkable malformation, the otic vesicle size was significantly smaller compared to that in the control fish at both 72 and 96 hpf (Figures 2A–C). Moreover, the *claudin h* morphants also showed obvious defects in both number and shape of the otoliths. Different from the wild type zebrafish who possessed a big otolith (saccular) and a small otolith (utricle), 83% of *claudin h* morphants either lost the utricle otolith or had an abnormal saccular otolith (Figures 2D–E).

### Deficiency of the *Claudin h* Caused Vestibular Dysfunction and Hearing Defects

**FIGURE 3:**
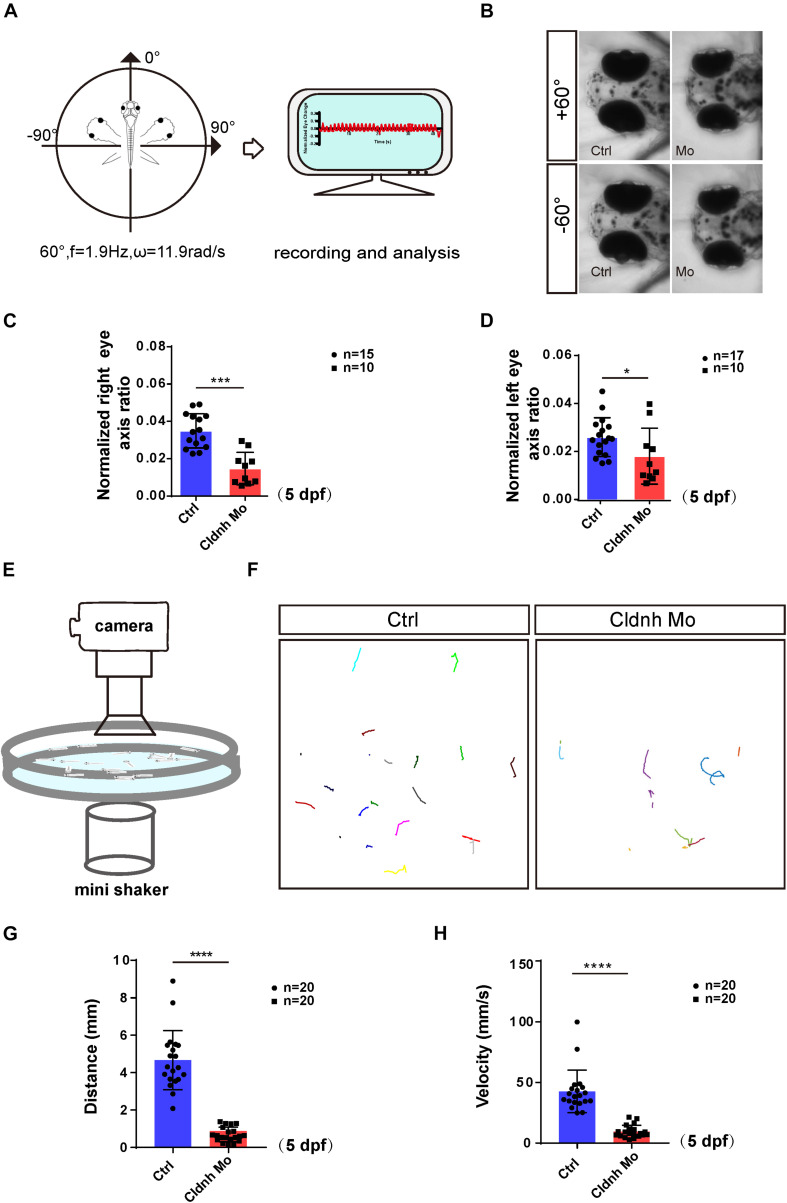
Loss function of the *claudin h* caused the vestibular dysfunction and hearing defects. **(A)** The schematic diagram shows the rotatory trajectory of the larva during VOR. **(B)** The heads and eyes of control and *claudin h* morphants acquired during VOR test at extreme tilting positions. **(C,D)** The vestibular function of zebrafish larvae at 5 dpf is evaluated by vestibular head tilt response measurement (right and left eyes, respectively). **(E)** The schematic diagram shows the startle response testing equipment. **(F)** The swimming trajectory of the control and *claudin h* morphants. **(G,H)** Swimming distance and peak velocity of zebrafish larvae at 5 dpf that reflected the auditory function of zebrafish larvae by examining the startle response. Values with *, ***, and ****above the bars are significantly different (*P* < 0.05, *P* < 0.001, and *P* < 0.0001, respectively).

To test whether abnormalities of the otic vesicles and otoliths in the *claudin h* morphants caused defects of balance perception or vestibular dysfunction, we tested VOR of AB zebrafish and *claudin h* morphants at 5 dpf using a customized VOR testing system (Figure 3A, Sun et al., 2018). The results showed that compared with the AB zebrafish who had robust eye movements, most of the *claudin h* morphants showed slight eye movements while rotating in the vertical plane in the machine (Figure 3B). The statistical analysis also showed that the amplitude of eye movements in *claudin h* morphants was significantly lower than that in AB zebrafish during VOR (*P* < 0.0001, Figures 3C,D).

To test whether malformation of the otic vesicles could also result in hearing dysfunction, a startle response experiment was performed. The results showed that the movement trajectory, swimming distance, and velocity of the *claudin h* morphants zebrafish larvae were significantly decreased compared to that of the controls at 5 dpf in startle responses (Figures 3E–H). These results indicated that the zebrafish hearing might be impaired by the *claudin h* Mo injection.

### Deficiency of the *Claudin h* Led to Decreased Hair Cells and Neuromasts

**FIGURE 4:**
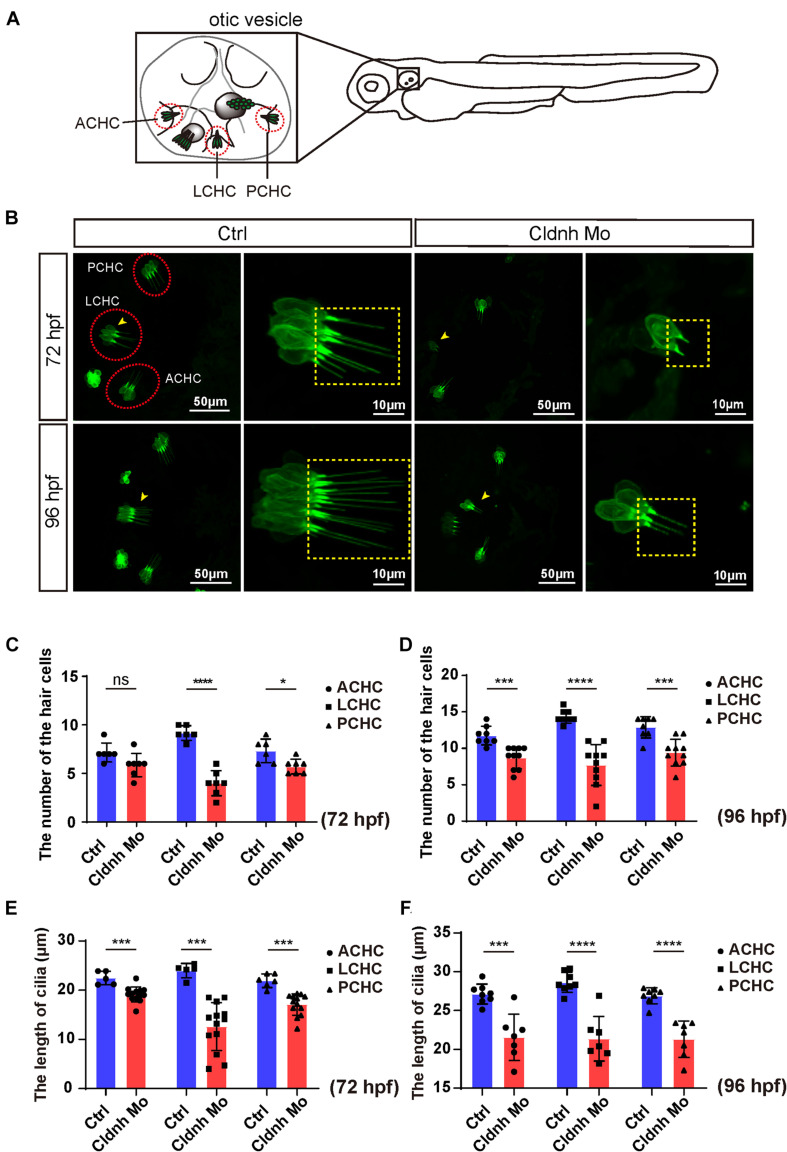
*Claudin h* deficiency suppressed cristae hair cells development. **(A)** The schematic for three different cristae hair cells in the otic vesicle. ACHC, anterior cristae hair cells; LCHC, lateral cristae hair cells; PCHC, posterior cristae hair cells. **(B)** Confocal imaging analysis of cristae hair cells in the otic vesicle of control and *claudin h* deficiency zebrafish at 72 and 96 hpf. The red dotted circle line marked the three different cristae hair cell clusters and magnified lateral cristae hair cell clusters (yellow arrow head) was shown in right and the yellow dotted square line marked the cilia of cristae hair cells. **(C,D)** The statistical analysis of the numbers of different cristae hair cells in the control and *claudin h* morphants at 72 and 96 hpf. **(E,F)** The statistical analysis of the cilia lengths of different cristae hair cells in the control and *claudin h* morphants at 72 and 96 hpf. Values with *, ***, and ****above the bars are significantly different (*P* < 0.05, *P* < 0.001, and *P* < 0.0001, respectively).

As we know, the hair cells in the inner ear of zebrafish are involved in balance perception and hearing. Therefore, to further investigate the cellular mechanisms of *claudin h* regulation on the ear function, morpholino-mediated gene knockdown was performed in the transgenic zebrafish line *Tg(Brn3c:mGFP)* where the hair cells are specifically labeled by GFP. The imaging results showed that although the three different cristae hair cell clusters, anterior cristae hair cells, lateral cristae hair cells, and posterior cristae hair cells still existed, the numbers of hair cells in each cluster were significantly decreased after the *claudin h* Mo injection at 72 and 96 hpf (Figures 4A–D). In addition, the growth of the cilia in the three different hair cell clusters was also affected by loss of *claudin h* and the statistical analysis demonstrated that the lengths of kinocilia in the *claudin h* morphants were significantly shorter than that in the control zebrafish at 72 and 96 hpf (Figures 4E,F).

**FIGURE 5:**
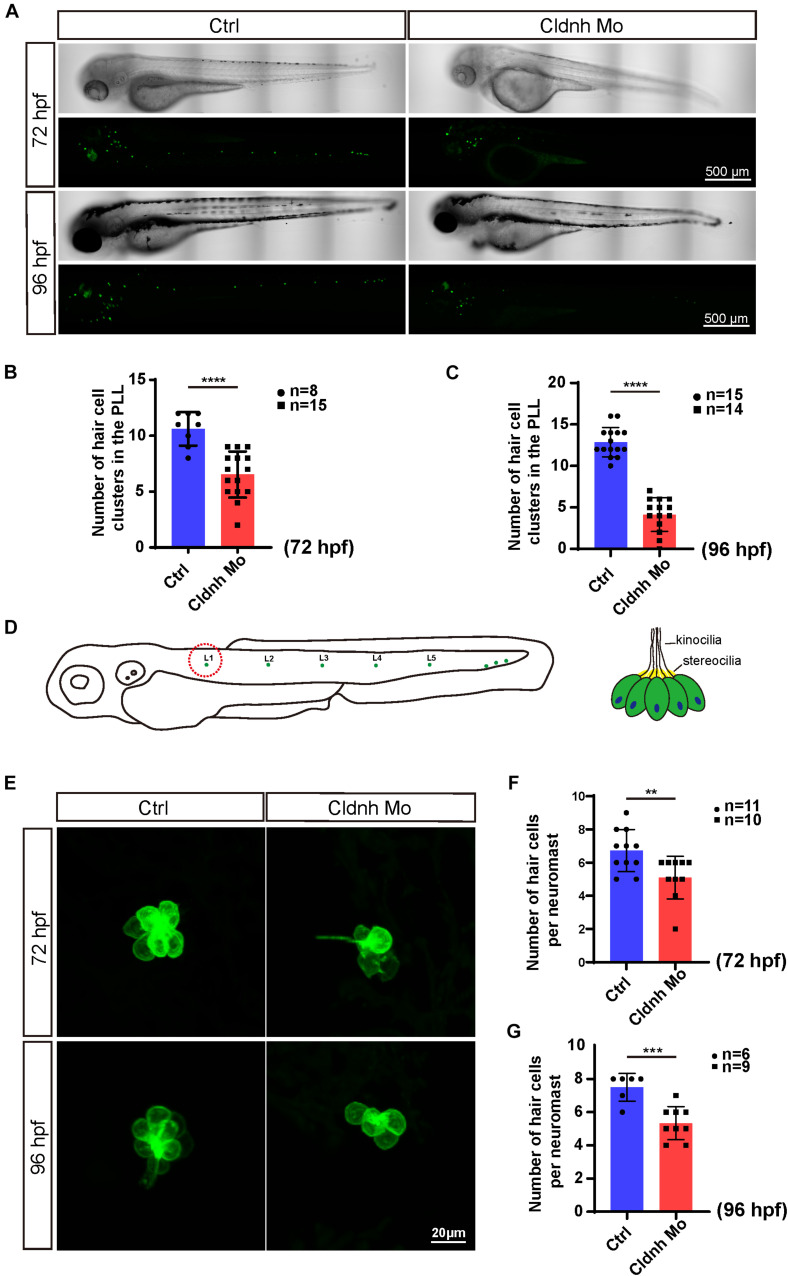
*Claudin h* knockdown decreased hair cell in the posterior lateral line of zebrafish. **(A)** The imaging analysis of control and *claudin h* morphants at 72 and 96 hpf in bright field and fluorescent field. Scale bar = 500 μm. **(B,C)** Quantification of the number of hair cell clusters in the posterior lateral line of control and *claudin h* morphants at 72 and 96 hpf. **(D)** The schematic for different hair cell clusters in the posterior lateral line. Scale bar = 10 μm. **(E)** Confocal imaging analysis of L1 hair cell clusters in the posterior lateral line of control and *claudin h* deficiency zebrafish at 72 and 96 hpf. **(F,G)** Quantification of the number of hair cells per L1 neuromast in the control and *claudin h* morphants at 72 and 96 hpf. Values with **, ***, and ****above the bars are significantly different (*P* < 0.01, *P* < 0.001, and *P* < 0.0001, respectively).

**FIGURE 6:**
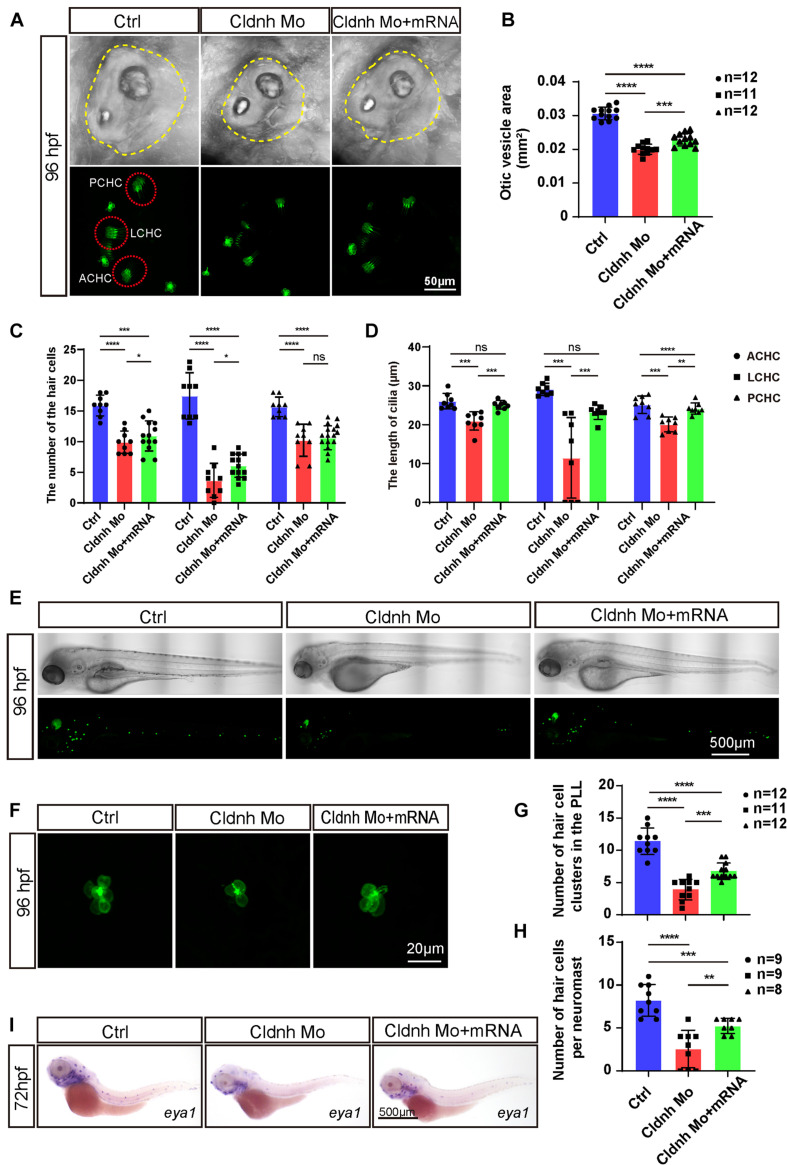
Overexpression of *claudin h* could rescue the development defects of hair cells in claudin morphants. **(A)** Imaging analysis of otic vesicle and cristae hair cells in control, *claudin h* morphants, and rescue group at 96 hpf. The yellow dotted line marked the boundary of the otic vesicle. Scale bar = 50 mm. **(B,C)** The statistical analysis of otic vesicle area in different groups at 96 hpf. **(C,D)** The statistical analysis of the number of different cristae hair cells and the cilia lengths in different groups at 96 hpf. **(E)** WISH results of the eya1 gene and the imaging analysis of control, *claudin h* morphants and rescue zebrafish at 96 hpf in bright field and fluorescent field. Scale bar = 500 μm. **(F)** Quantification of the number of hair cell clusters in the posterior lateral line of different groups at 96 hpf. **(G)** Confocal imaging analysis of L1 hair cell clusters in the posterior lateral line of different groups at 96 hpf. **(H)** Quantification of the number of hair cells per L1 neuromast in the control, *claudin h* morphants, and rescue zebrafish at 96 hpf. Values with *, **, ***, and ****above the bars are significantly different (*P* < 0.05, *P* < 0.01, *P* < 0.001, and *P* < 0.0001, respectively). **(I)** At 72 hpf, the *in situ* hybridization signal of *claudin h* in the control zebrafish, claudin h morphants, and rescue zebrafish.

The WISH results showed that *claudin h* was also localized in the neuromasts of the trunk. As we know the hair cells in the lateral line are crucial for perceiving changes in the surroundings. Therefore, we further detected whether *claudin h* was also involved in the formation of neuromasts in the trunk. Interestingly, the results showed that the number of hair cell clusters in the posterior lateral line of *claudin h* morphants were remarkably reduced (Figures 5A–C). Besides, in order to detect whether hair cells in the remaining clusters were affected, we further imaged and counted the number of L1 hair cells and found that it was significantly decreased after the morpholino injection at 72 and 96 hpf (Figures 5D–G). Moreover, we also performed WISH using the probe of eya1 gene, which was reported to be localized in neuromasts of zebrafish (Qian et al., 2020), and the result indicated that the loss of the *claudin h* could also reduce the numbers of neuromasts in the lateral line of zebrafish (Figure 6I).

### The Reduction of Hair Cells in the *Claudin h* Deficient Zebrafish Was Caused by Cell Apoptosis

**FIGURE 7:**
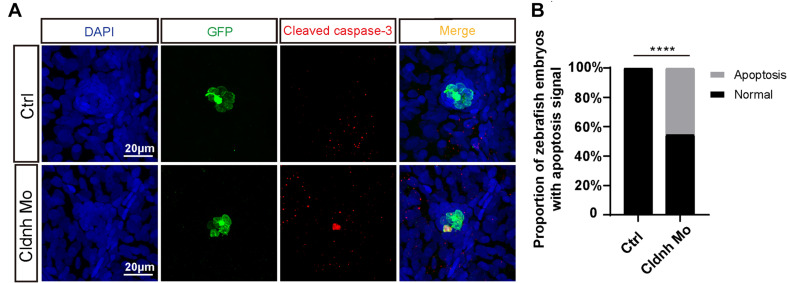
*Claudin h* deficiency caused the hair cells apoptosis. **(A)** DAPI and cleaved caspase-3 staining for the L1 hair cell clusters in the posterior lateral line of the control zebrafish and *claudin h* morphants. Scale bar = 10 μm. **(B)** Quantification of zebrafish embryos with the hair cell apoptosis in the control and *claudin h* morphants. Values with **** above the bars are significantly different (*P* < 0.0001).

To investigate cellular mechanism for the loss of the hair cells, immunohistochemistry assay was performed on the *claudin h* deficient and control zebrafish at 72 hpf with the antibody against cleaved caspase-3, one of the apoptosis markers. In the results, though very little signal was detected in controls, much more cleaved caspase-3-positive cells emerged and overlapped with the hair cells in the *claudin h* morphants (Figure 7), indicating the lack of hair cells might be caused by the hair cells apoptosis.

### The Defective Phenotype of the *Claudin h* Morphants Could Be Rescued by *Claudin h* mRNA

In order to confirm that the hair cell morphological defects were specifically caused by the *claudin h* deficiency, we co-injected the *in vitro* synthesized *claudin h* mRNA containing an intact open reading frame with *claudin h* Mo into one cell stage zebrafish embryos to test whether exogenous *claudin h* could rescue the phenotype that was found in the *claudin h* morphants. Interestingly, the *claudin h* mRNA co-injection could partially rescue the abnormal phenotypes including the smaller size of the otic vesicle and the reducing number of hair cells in the otic vesicle compared to the *claudin h* morphants (Figures 6A–D). Similarly, both the number of hair cell clusters in the lateral line and in each L1 cluster were also partially recovered after the *claudin h* mRNA coinjection (Figures 6E–H). Taken together, these results indicated that those otic vesicle and hair cell defects found in this study were specifically caused by loss of *claudin h*.

### CRISPR/Cas9-Mediated *Claudin h* Mutation Disrupts Hair Cell Development

In order to further validate the function of *claudin h* during the hair cell development, the CRISPR/Cas9 system was utilized to knockout *claudin h* in *Tg(Brn3c:mGFP)* transgenic zebrafish. As shown in Supplementary Figure 3A, we chose a sgRNA target site near the translation start codon (ATG) in the exon1 of *claudin h* for CRISPR/Cas9-mediated mutation to abolish the protein translation. The mutations were successfully induced into the targeting site which was verified by PCR and sequencing (Supplementary Figure 3B).

**FIGURE 8:**
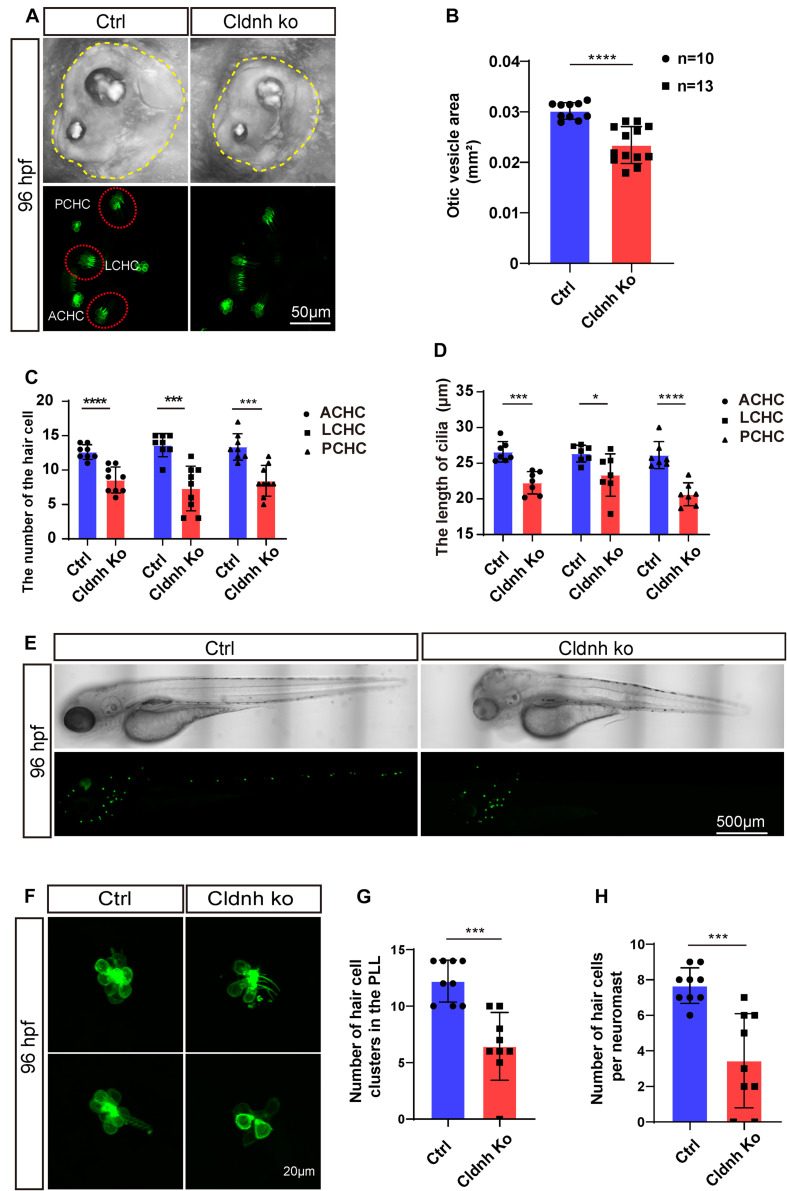
Knocking out of *claudin h* caused the defects of otic vesicle and impaired the development of cristae hair cells and neuromast hair cells. **(A)** Imaging analysis of otic vesicle and cristae hair cells in control and *claudin h* mutants at 96 hpf. The yellow dotted line marked the boundary of the otic vesicle. **(B)** The statistical analysis of otic vesicle area in control and *claudin h* mutants at 96 hpf. **(C,D)** The statistical analysis of the number of different cristae hair cells and the cilia lengths in control and *claudin h* mutants at 96 hpf. **(E)** The imaging analysis of control and *claudin h* mutants at 96 hpf in bright field and fluorescent field. **(F)** Confocal imaging analysis of L1 hair cell clusters in the posterior lateral line of control and *claudin h* mutants at 96 hpf. **(G,H)** Quantification of the number of hair cell clusters and the number of hair cells per L1 neuromast in the posterior lateral line of control and *claudin h* mutants at 96 hpf. Values with *, ***, and ****above the bars are significantly different (*P* < 0.05, *P* < 0.001, and *P* < 0.0001, respectively).

Similar to the results of the *claudin h* morphants, the otic vesicle size of the *claudin h* mutants was remarkably smaller than that of the control fish at 96 hpf (Figures 8A,B). Meanwhile, the number of hair cells in the anterior cristae, lateral cristae, and posterior cristae was significantly decreased and the lengths of the hair cell cilia were shortened as well in the *claudin h* mutant zebrafish at 96 hpf (Figures 8C,D). Moreover, we also found that the number of hair cell clusters and the number of hair cells in each neuromast in the lateral line were also decreased significantly after knocking out of *claudin h*, which was consistent with the *claudin h* morphants (Figures 8E–H).

## Discussion

It is well known that tight junction strands composed of claudins are crucial for the function of permeability barrier of the emunctory like the kidney. However, more and more studies found that many *claudins*, such as *claudin a*, *claudin b*, *claudin j*, *claudin 7b*, *claudin 9*, and *claudin14*, are expressed in the inner ear and necessary for its development, implying that tight junctions in the inner ear might also contain multiple claudins to tightly seal the different regions to regulate the function of the ear including hearing and balance perception during the development (Ben-Yosef et al., 2003; Hardison et al., 2005; Nakano et al., 2009; Li et al., 2018). In this study, the *claudin h* was also found to express in the otic vesicle and the lateral line neuromast, indicating *claudin h* might be also involved in the formation and function of otic vesicle and lateral line.

The development of otic vesicle and formation of otoliths were clearly impaired in the zebrafish lacking *claudin h* in this study. Similarly, it has been reported that though the deficiency of *claudin j* did not directly affect the generation of the calcium carbonate and protein complex, it remarkably prevented these crystallites from efficiently aggregating into the otoliths (Hardison et al., 2005). Moreover, *claudin 7b* was also reported to regulate the formation of otoliths in zebrafish (Li et al., 2018). As we know claudins are necessary for the tight junctions to separate the extracellular fluids by sealing off the paracellular spaces, and many studies have shown that altering or removing the claudins in the tight junctions can alter the ionic permeability barrier (Furuse et al., 2001; Colegio et al., 2003). During the development of zebrafish, the otic vesicle gradually develops into a well-balanced ionic and fluid-filled vesicle with compositional division of the distinct paracellular fluids with tight junctions and thereby to support the normal development of hair cells and otoliths (Li et al., 2018). Therefore, the otolith phenotype might be due to the loss of *claudins* which disrupted the ionic composition in the otic vesicle and further prevented the fusion of crystallites, which was indispensable for normal otolith formation (Hardison et al., 2005).

Otoliths can transmit acoustic vibrations and acceleration forces to the hair cells. The utricular otolith is indispensable for vestibular function, while saccular otoliths are only necessary for hearing (Popper and Fay, 1993; Riley and Moorman, 1999). It has been reported that in zebrafish, *claudin j* expressed in the otic placode and loss of function of *claudin j* caused severe reduction in otoliths size, insensitivity to tapping stimulus, and inability to control balance, which indicated that the hearing and vestibular function were significantly affected by the deficiency of *claudin j* (Hardison et al., 2005). Nowadays, eyes movement detected by VOR have been used to assess the vestibular function. In this study, significant vestibular dysfunction was also detected in the *claudin h* morphants by VOR, which was consistent with the loss of the utricle otolith in *claudin h* morphants. In addition, the startle response of zebrafish that has been used as a reliable evaluation of hearing (Yang et al., 2017) was also performed in this study, and revealed an auditory defect in the *claudin h* morphants (Figures 3F–H). Overall, these results clarified that the loss of *claudin h* could lead to the defects of otic vesicle and otolith, and further result in vestibular dysfunction and auditory handicap.

Sensory hair cells in the membranous labyrinth of otic vesicle and lateral line neuromasts in zebrafish serve as the receptors for the acoustic signals and pressure acceleration in the surrounding area which then transform these signals or mechanical pressure into chemical or electrical signals to activate auditory or vestibular circuitry (Baxendale and Whitfield, 2016). A large number of studies have shown that hair cell damage is a leading cause for hearing loss (He et al., 2016; Yu et al., 2017; Zhang et al., 2019). Therefore, investigating whether the development and formation of the hair cells were affected by the *claudin h* deficiency could further illustrate the potential cellular mechanisms of the vestibular and auditory dysfunction found in this study. It has been reported that *claudin14* was expressed in the outer hair cells, cochlear and supporting cells in mice, and the deletion of this gene would lead to significant degeneration of both cochlear outer hair cells and inner hair cells which further cause the congenital hearing deficiency during the first 3 weeks of life (Ben-Yosef et al., 2003). Similarly, we also found the hair cell numbers were remarkably decreased in both otic vesicle and lateral line of *claudin h* morphants and mutants, though most of the hair cell clusters still existed. Moreover, the growth of the hair cell kinocilia was also significantly suppressed by the defects of *claudin h* in this study. These results indicated that the abnormal morphogenesis and formation of hair cells might be a crucial reason for the inner ear dysfunction. Nowadays, many *claudin* genes, such as *claudin a*, *claudin b*, *claudin 7b*, *claudin f*, and *claudin j*, have been reported to be expressed in otic vesicle or lateral line neuromasts during zebrafish larvae development^1^ and mutation of them might cause different developmental defects. For example, *claudin 7b* was indispensable for the otic epithelial structure, the otolith formation and sound stimulation sensitiveness by sustaining initial integrity of otic epithelia during embryogenesis; nevertheless, it had no impact on the number and morphology of hair cells (Li et al., 2018). Moreover, although similar otolith phenotype and hearing defects were also found in the *claudin j* mutants, the epithelial structure of the otocyst does not seem to be protruding disrupted (Hardison et al., 2005). Taken together, these results indicated that multiple *claudins* are involved and properly orchestrated to regulate the development and function of the otic vesicle during zebrafish larvae development.

In this study, apoptosis analysis results showed that more cleaved caspase-3-positive cells that were colocalized with the hair cell emerged in the *claudin h* morphants, indicating the lack of hair cells was caused by the hair cell apoptosis. Apoptosis is one of the common pathways for cell death which was characterized by DNA degradation (Morrill and He, 2017). Nowadays, apoptosis is also considered to be one of the mechanisms for the sensory hair cell death induced by acoustic trauma and the caspase-3 activation is a key step during this process (Qian et al., 2020). As the most important components of the tight junctions, claudins play a central role in regulation of paracellular permeability and could create charge-selective channels in the paracellular space with varied combinations or expression levels of claudins (Ben-Yosef et al., 2003). It has been reported that claudin 9 is necessary to form a paracellular ion permeable barrier for Na^+^ and K^+^. Knocking out of the *claudin 9* in mice would eliminate this ion barrier function and thereby increase the K^+^ concentration in the basolateral fluid of the hair cells (Nakano et al., 2009). Similarly, *claudin 14* mutation was also reported to induce the hair cell degeneration by altering ionic permeability of the paracellular barrier for K^+^ to further cause human deafness (Ben-Yosef et al., 2003). Besides, longtime exposure in high concentration of K^+^ was significantly toxic to hair cells and could suppress hair cell repolarization which eventually led to the hair cells death (Hibino and Kurachi, 2006). Altogether, we hypothesize that the deficiency of *claudin h* in the otic vesicle might have changed the ionic composition through the disruption of paracellular ionic permeability which then induced the hair cell apoptosis and further caused the hearing loss and vestibular dysfunction. However, more details need to be confirmed in future studies. In summary, our characterization of the *claudin h* morphants and mutants not only revealed the biological significance of *claudin h* for the development and function of the hearing organs, but also provided new insights into the pathogenesis of human deafness due to loss of claudin proteins.

## Data Availability Statement

The datasets presented in this study can be found in online repositories. The names of the repository/repositories and accession number(s) can be found in the article/Supplementary Material.

## Ethics Statement

The animal study was reviewed and approved by Administration Committee of Experimental Animals, Jiangsu Province, China.

## Author Contributions

DL conceived the project. JG, PQ, YH, CG, CW, CC, and HW performed most of the experiments. DL, JG, and HW analyzed the data and prepared the manuscript. All authors commented and approved the manuscript.

## Conflict of Interest

The authors declare that the research was conducted in the absence of any commercial or financial relationships that could be construed as a potential conflict of interest.

## Footnotes

1 http://ZFIN.org

## Supplementary Material

Supplementary Figure 1 *Claudin h* expression analyses in the otic vesicle and hair cells. **(A)** At 24 hpf, the *in situ* hybridization signal of *claudin h* is localized in the otic vesicle, posterior lateral line primordium, and pronephros. **(A’)** The magnified figure of the region squared in dashed line. **(B–D)** *Claudin h* expressed in the in the otic vesicle and neuromast.

Supplementary Figure 2 The verification of *fgfbp3* Mo efficiency. **(A)** Schematic diagram for the transcription after fgfbp3 Mo injection. **(B)** The result of RT-PCR of wild type zebrafish and *fgfbp3* morphants.

Supplementary Figure 3 Generation of zebrafish *claudin h* mutant using CRISPR/Cas9 system. **(A)** Schematic diagram showing the targeting site of the sgRNA on the first exon of *claudin h* gene. **(B)** Mutations occurred in the target site of the *claudin h* gene in mutant zebrafish compared to the wild-type fish.

Supplementary Sequences The sequencing results for *claudin h* mutant and wildtype zebrafish.
