# Supplementary figures and images for: Claudin h Is Essential for Hair Cell Morphogenesis and Auditory Function in Zebrafish
Source: Front Cell Dev Biol. 2021 May 11;9:663995. doi: 10.3389/fcell.2021.663995 (PMC8147561; doi:10.3389/fcell.2021.663995)

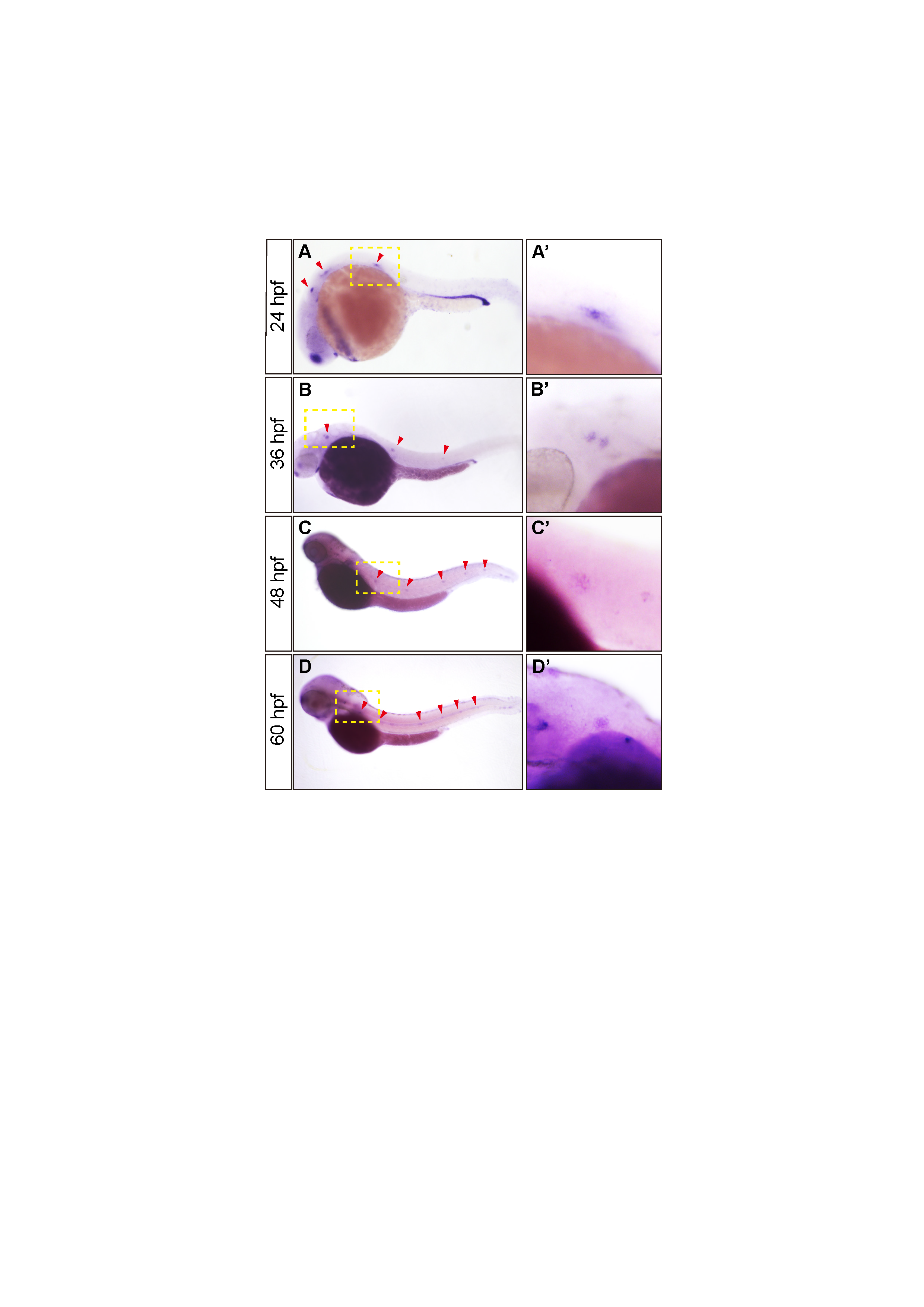

Supplement: Supplementary Figure 1 — Claudin h expression analyses in the otic vesicle and hair cells. (A) At 24 hpf, the in situ hybridization signal of claudin h is localized in the otic vesicle, posterior lateral line primordium, and pronephros. (A’) The magnified figure of the region squared in dashed line. (B–D) Claudin h expressed in the in the otic vesicle and neuromast. [file Image_1.TIF]

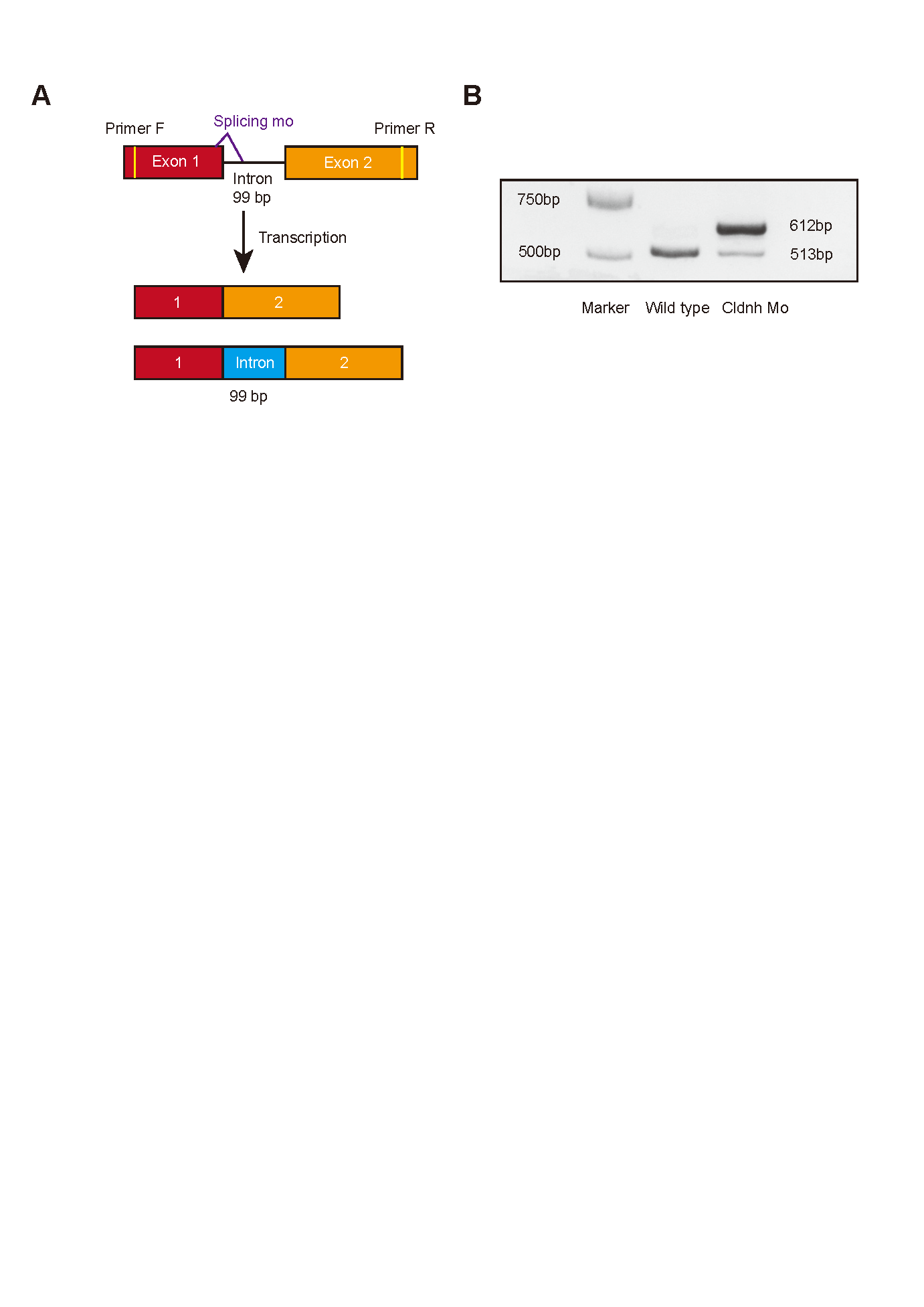

Supplement: Supplementary Figure 2 — The verification of fgfbp3 Mo efficiency. (A) Schematic diagram for the transcription after fgfbp3 Mo injection. (B) The result of RT-PCR of wild type zebrafish and fgfbp3 morphants. [file Image_2.TIFF]

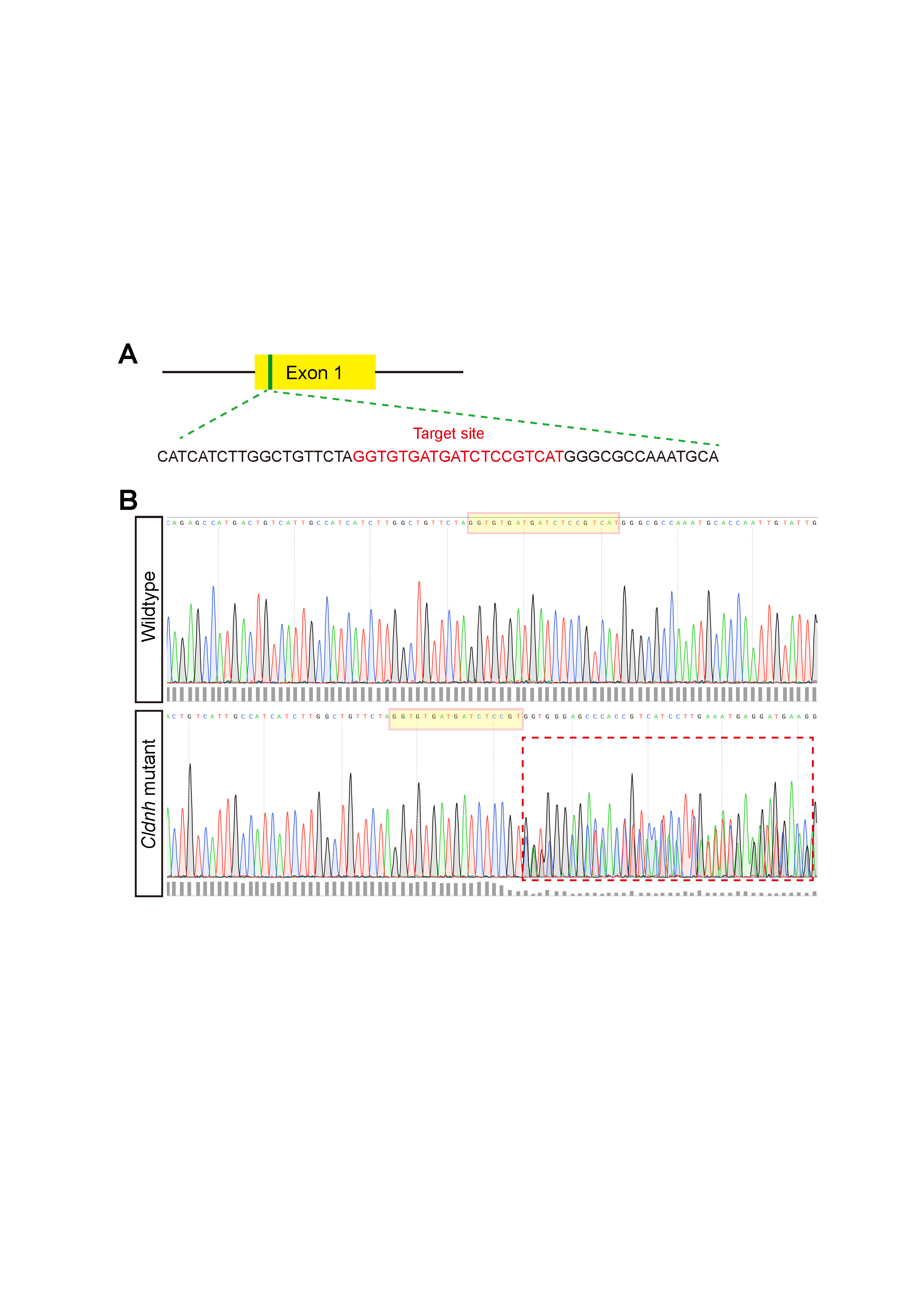

Supplement: Supplementary Figure 3 — Generation of zebrafish claudin h mutant using CRISPR/Cas9 system. (A) Schematic diagram showing the targeting site of the sgRNA on the first exon of claudin h gene. (B) Mutations occurred in the target site of the claudin h gene in mutant zebrafish compared to the wild-type fish. [file Image_3.TIF]
