# Supplementary material for: Claudin h Is Essential for Hair Cell Morphogenesis and Auditory Function in Zebrafish
Source: Front Cell Dev Biol. 2021 May 11;9:663995. doi: 10.3389/fcell.2021.663995 (PMC8147561; doi:10.3389/fcell.2021.663995)
Supplement: Supplementary Sequences — The sequencing results for claudin h mutant and wildtype zebrafish. [file Table_1.DOCX]

***Claudin h* mutant：**

GTATGTGGGATGGTTTATGGATGAACTGTGTCGTTCAGAGCACTGGACAGATGCAGTGTAAGGTCTACGACTCAATGCTGGCTTTAGGTCAAGATCTGCAGGCGTCCAGAGCCATGACTGTCATTGCCATCATCTTGGCTGTTCTAGGTGTGATGATCTCCGTGGTGGGAGCCCACCGTCATCCTTGAAATGAGGATGAAGGTGCGAATGGAAGAGGGATGAGCTACTCTGAAATCGAGGACCTTATCGGTGATCTCGTGCATTATTGTCCTTCTCCCGGATCGCATTCCCTCATCCTGCGGGACTTTTACAACCATTCACTGCCCTTACCTCCGATACAGACCTCGGCTCATCCATTAAAGTACGTTTCATTGCTGTACCTCTCTTACTCTGCGGACGACCTCTGATGTATTGGAGGACCCATGCTGTA

**Wildtype zebrafish:**

CGCAAGTAATGTGGGATGGTTTATGGATGAACTGTGTCGTTCAGAGCACTGGACAGATGCAGTGTAAGGTCTACGACTCAATGCTGGCTTTAGGTCAAGATCTGCAGGCGTCCAGAGCCATGACTGTCATTGCCATCATCTTGGCTGTTCTAGGTGTGATGATCTCCGTCATGGGCGCCAAATGCACCAATTGTATTGAGGATGAAGGTGCTAAGGCTAAAGTGATGATCGTCTCTGGCATCATGTTCATTATTGCTGGCATCCTGGATCTCATTCCTTCAGCCTGGGTGGCAAACCAGATCATTCGGGACTTTTACAACCCGTTACTGCCCGGCGCTCAGCAAAGAGAGCTCGGGGCATCCATTTACATAGGTTTCGCTGCTGCCGCTCTCTTAATCATTGGAGGACCCATGCTGTTACA
